# The learning curve of V-NOTES hysterectomy: a single-surgeon experience

**DOI:** 10.3389/fmed.2025.1574457

**Published:** 2025-06-18

**Authors:** François Charles, Mathieu Luyckx, Nathanael Dubois

**Affiliations:** ^1^Saint Jean Clinic, Brussels, Belgium; ^2^Cliniques Universitaires Saint-Luc, Brussels, Belgium

**Keywords:** hysterectomy, natural orifice transluminal endoscopic surgery, V-NOTES, learning curve, CUSUM analysis

## Abstract

**Purpose:**

Vaginal natural orifice transluminal endoscopic surgery (V-NOTES) has emerged as an innovative surgical approach that combines the advantages of endoscopic surgery with those of the vaginal approach. This article presents the initial experience of a single institution in implementing the V-NOTES technique for hysterectomy procedures.

**Methods:**

This retrospective cohort study assessed the first 139 V-NOTES hysterectomies performed by a single surgeon at 000 from 2021 to 2024. Preoperative, intraoperative, and postoperative data were recorded. Operative time (OT) served as an indicator of surgical proficiency. The learning curve was analyzed using the cumulative sum method of operative time (CUSUMot).

**Results:**

The mean OT was 59.32 ± 21.13 min. No patient required conversion to laparotomy or conventional laparoscopy. The CUSUMot analysis demonstrated four phases in the learning curve: initiation (12 patients; mean OT, 66.17 ± 12.84 min), consolidation (41 patients; mean OT, 54.32 ± 13.96 min), complexification (54 patients; mean OT, 64.46 ± 25.48 min), and mastery (32 patients; mean OT, 54.5 ± 21.11 min). Additionally, the study identified a history of cesarean section and the absence of vaginal delivery as two risk factors for bladder injury.

**Conclusion:**

This study identifies a four-phase learning curve for V-NOTES hysterectomy, with initial competency achieved after 12 cases and sufficient mastery reached after 53 cases for an experienced surgeon.

## Introduction

Hysterectomy, one of the most common benign surgical procedures in gynecology, has undergone considerable advancements over the years owing to technological innovations and new surgical approaches. In 2023, a Cochrane review recommended using a vaginal hysterectomy (VH) approach whenever feasible. When a VH is not achievable, laparoscopic hysterectomy (LH) offers several advantages compared to abdominal hysterectomy (AH), although it has a higher risk of ureteral injuries. Notably, evidence regarding vaginal natural orifice transluminal endoscopic surgery (V-NOTES) hysterectomy remains limited (1).

V-NOTES hysterectomy, first described in 2012 (2), is gradually becoming an innovative and less invasive alternative to traditional techniques, combining the advantages of endoscopic surgery with a transvaginal approach. Since its introduction, numerous teams have established its feasibility and safety (2, 3), even for large-volume uteri (4). Compared to LH, the HALON study revealed the non-inferiority of V-NOTES regarding conversions, postoperative infections, perioperative complications, and hospital readmissions within 6 weeks, and its superiority in operative time (OT) and hospital stay (5). These findings have been substantiated by a recent systematic review (6) and meta-analysis (3), which also reported lower rates of blood transfusion, postoperative complications, and a reduced visual analog scale (VAS) pain score.

In a series of 750 V-NOTES hysterectomies, the overall complication rate was 5.2%, comprising 1.4% perioperative complications—primarily bladder injuries—and 3.8% postoperative complications, including lower urinary tract infections, hematomas, nausea, vomiting, and wound infections (7). Since its implementation in our institution in April 2021, over 150 V-NOTES hysterectomies have been performed. This article aims to share our team’s experience in adopting the V-NOTES technique for hysterectomies, emphasizing the learning curve observed during the first 139 cases performed by a single surgeon.

## Methods

In this retrospective cohort study, we collected data from the first 139 V-NOTES hysterectomies performed by a single gynecologic surgeon at our institution between April 2021 and June 2024.

### Training

The primary surgeon, possessing over a decade of extensive experience in LH, underwent dedicated theoretical training on the V-NOTES technique prior to its integration into clinical practice in April 2021.

### Procedure

The V-NOTES hysterectomy procedure followed the standardized 10-step approach described in Housmans et al. (8):

1. Circumcision of the cervix following infiltration with a mixture of local anesthesia and adrenaline.
2. Posterior colpotomy.
3. Anterior colpotomy.
4. Transection of the uterosacral ligaments.
5. Preparation and placement of the V-NOTES port.
6. Identification of the ureters and transection of the parametrium.
7. Transection of the infundibulopelvic or utero-ovarian ligament.
8. Hemostasis and port removal.
9. Specimen removal.
10. Vault closure.

All hysterectomies employed the GelPOINT V-Path PLATFORM (Applied Medical, C2A12), using a laparoscopic bipolar coagulation clamp and a 5 mm VOYANT fusion device (Applied Medical, EB210) as laparoscopic instruments. All patients received 2 g of cefazolin and 1.5 g of metronidazole at induction, followed by an additional 2 g dose of cefazolin 8 h after the procedure. Owing to institutional constraints, the standardized approach’s recommendation for vaginal application of clindamycin cream 2 h prior to surgery was not implemented.

Patient selection for the V-NOTES procedure was based on contraindications, including a history of rectal surgery, pelvic radiation therapy, suspected rectovaginal endometriosis, pelvic inflammatory disease (PID), active infection, virginity, and pregnancy. In 2021, an expert consensus recommended selecting initial cases based on a BMI limit of 30, a small uterine size (<11 cm), and no history of cesarean section (9). However, in our institution, any patient eligible for LH without contraindications underwent V-NOTES hysterectomy without further selection because our practice commenced before the publication of this consensus. This study was performed with the approval of the ethics committee regarding protocol and data collection.

### Data collection and statistical analysis

Data were collected retrospectively from electronic medical records, encompassing preoperative parameters (age, BMI, surgical history, obstetric history, and surgical indication), perioperative details (OT from cervical infiltration to vaginal closure, perioperative complications, hospital stay duration, quantity of level 2–3 analgesics administered during hospitalization, and weight of the anatomopathologic specimen after fixation), and postoperative outcomes (immediate and late complications, as well as readmissions in 6 weeks).

To evaluate the learning curve, we used the cumulative sum of operative time (CUSUMot) method, a widely used calculation method in comparable studies (10–12). The CUSUMot method allows us to visualize this curve, demonstrating the evolution of OT as the number of procedures performed increases. The CUSUM is calculated by accumulating deviations from the mean and is sensitive to performance changes. This allows for the detection of improvements or declines in the surgeon’s proficiency as they gain experience. The calculation is as follows. The CUSUMot of the first case is the difference between the OT of the first case and the mean of all OTs. The CUSUMot of the second case is calculated by adding the CUSUMot of the first case to the difference between the OT of the second case and the mean OT of all cases. This process is repeated for each subsequent case until the last case, at which point the CUSUM reaches zero.

All statistical analyses were performed using RStudio. Owing to the non-normal distribution of our variables, the Kruskal–Wallis test was used to compare continuous variables. Categorical variables were analyzed using chi-square tests or Fisher’s exact tests. All analyses were two-tailed, with *p* < 0.05 considered statistically significant. In instances where the Kruskal–Wallis test revealed a significant difference, *post-hoc* analysis was performed according to Dunn’s test to perform pairwise comparisons between groups.

## Results

Table 1 presents the patient characteristics and perioperative and postoperative data. Between April 2021 and June 2024, 139 patients underwent hysterectomy with bilateral salpingectomy or oophorectomy via V-NOTES without conversion to laparoscopy or laparotomy. The mean OT, measured from cervical incision to vaginal closure, was 59.32 ± 21.13 min. The only perioperative complications encountered were bladder injuries sustained during the anterior colpotomy. These injuries were identified and addressed during the procedure through the vaginal approach. Subsequently, an indwelling urinary catheter was placed for 7–10 postoperative days, followed by a cystography upon removal, which showed no bladder wall defects. At the postoperative check-up 6 weeks after the procedure, all patients exhibited satisfactory urination.

**Table 1:** Characteristics, perioperative and postoperative data^a^.

Variable	Value
*N*	139
Age (years)	48.98 ± 27.66
BMI (kg/m^2^)	27.66 ± 5.19
History of pregnancy	121 (87.1%)
History of vaginal delivery	100 (71.9%)
History of cesarean section	26 (18.7%)
History of laparoscopy	57 (41%)
History of laparoscopy	1 (0.7%)
Operative indications	
Myomatosis	70 (50.4%)
Adenomyosis	23 (16.5%)
Endometrial pathology	23 (16.5%)
Oncological prophylaxis	4 (2.9%)
Cervical dysplasia	3 (2.2%)
Mixed^b^	16 (11.5%)
Operative time (minutes)	59.32 ± 21.13
Intraoperative complication	5 (3.6%)
Cystotomy	5 (3.6%)
Conversion	0
Postoperative stay duration (days)	1.19 ± 0.5
Number of grade 2–3 analgesics	0.60 ± 0.84
Postoperative complications	8 (5%)
CD I–II^c^	
Lower urinary tract infection	2 (1.4%)
Ileus	2 (1.4%)
Vaginal granuloma	1 (0.7%)
Pneumonia	1 (0.7%)
Infected hematoma	1 (0.7%)
CD III	
Hemoperitoneum	1 (0.7%)
CD IV	0
Readmissions within 6 weeks	2 (1.4%)
Weight of the pathology specimen after fixation (gram)	258.42 ± 219.19

a Data are presented as mean ± standard deviation or as number (percentage).

b Mixed indications, including associations of adenomyosis and myomatosis, ovarian cysts and dysmenorrhea.

c Classification of postoperative complications according to the Clavien–Dindo classification.

Postoperative complications observed were classified according to the Clavien–Dindo classification (13). Among the 139 patients, two experienced lower urinary tract infections confirmed by urine culture and treated with antibiotics. One of these patients had a perioperative bladder injury and an indwelling urinary catheter for 10 postoperative days. Two patients developed ileus, requiring readmission. One was managed with antiemetics, while the other required nasogastric decompression. A single patient experienced vaginal suture site granuloma, leading to metrorrhagia. This bleeding was controlled as an outpatient following silver nitrate application. Postoperatively, one patient developed pneumonia and was treated with antibiotics on an outpatient basis. Another patient experienced a superinfection of a hematoma at the vaginal dome, which was managed as an outpatient with antibiotics. Finally, one patient developed hemoperitoneum on postoperative day 1, requiring laparoscopic drainage of the hemoperitoneum and transfusion of one unit of red blood cells. Notably, no active bleeding was identified at the surgical site in this patient.

**Figure 1:**
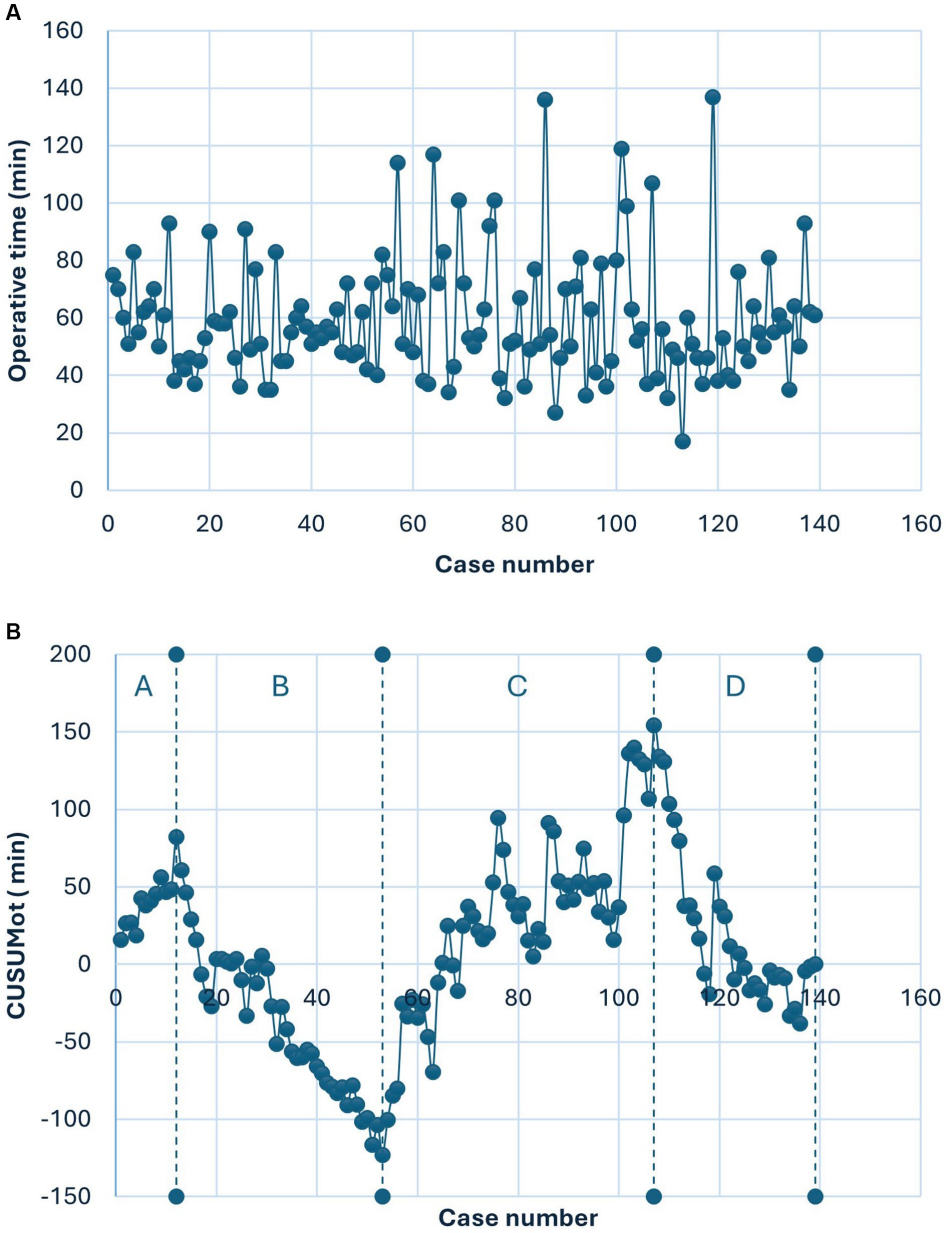
**(A)** Shows the relationship between operative time and the number of cases in chronological order. **(B)** Shows the relationship between CUSUMot and the number of cases in chronological order.

In terms of the learning curve, Figure 1A shows the chronological correlation between OT and case count. Figure 1B shows the CUSUM method of OT, revealing four distinct phases. Phase A, encompassing cases 1–12, represents the initiation phase. The surgeon first performed V-NOTES hysterectomies on patients without specific criteria; BMIs varied up to 35.9, uterine weights ranged from 72 to 403 g, and one patient had a prior cesarean section. The average OT for this phase was 66.17 ± 12.84 min. Phase B, comprising cases 13–53, is the consolidation phase. During this period, the surgeon and surgical team gradually gained proficiency with the technique. A trend of decreasing OTs was evident, reflecting improved surgical proficiency. The average OT for this phase was 54.32 ± 13.96 min. Phase C (cases 54–107) represents the complexity phase, during which OT tended to increase. The average OT for this phase was 64.46 ± 25.48 min. Phase D (cases 108–139) marks the mastery phase, with an average OT of 54.5 ± 21.11 min.

Table 2 presents a summary of group characteristics, perioperative data, and postoperative outcomes according to the four phases of the learning curve. Comparative analysis of the four phases revealed no statistically significant differences in terms of BMI, obstetric history, surgical history, perioperative and postoperative complications, length of hospital stay, number of analgesics used, readmission in 6 weeks, or specimen weight.

**Table 2:** Characteristics, perioperative, and postoperative data of the four phases^a^.

Variable	Phase A	Phase B	Phase C	Phase D	*p*^b^
*N*	12	41	54	32	
Age (years)	45.92 ± 5.35	46.56 ± 7.29	51.46 ± 9.28	49.03 ± 7.51	0.015
BMI (kg/m^2^)	28.23 ± 3.71	27 ± 4.63	28.34 ± 6.09	26.81 ± 4.55	0.436
History of pregnancy	11 (91.7)	35 (85.4)	46 (85.2)	29 (90.6)	0.914
History of vaginal delivery	10 (83.3)	31 (75.6)	35 (64.8)	24 (75.0)	0.522
History of cesarean section	1 (8.3)	9 (22.0)	13 (24.1)	3 (9.4)	0.298
History of laparoscopy	7 (58.3)	18 (43.9)	19 (35.2)	13 (40.6)	0.487
History of laparotomy	0 (0.0)	0 (0.0)	0 (0.0)	1 (3.1)	0.317
Operative time (minutes)	66.17 ± 12.84	54.32 ± 13.96	64.46 ± 25.48	54.5 ± 21.11	0.027
Intraoperative complications	0	0	3 (5.6)	2 (6.3)	0.366
Postoperative stay duration (days)	1.08 ± 0.29	1.34 ± 0.75	1.15 ± 0.36	1.09 ± 0.30	0.296
Number of grade 2–3 analgesics	0.67 ± 0.89	0.68 ± 0.88	0.56 ± 0.79	0.53 ± 0.88	0.784
Postoperative complications	0	5 (12.2)	3 (5.6)	0	0.130
Readmissions within 6 weeks	0	1 (2.4)	1 (1.9)	0	1.000
Weight of the pathology specimen after fixation (gram)	206.08 ± 102.08	214.21 ± 143.49	304.89 ± 282.59	256.13 ± 197.68	0.744

a Data are presented as mean ± standard deviation or as number (percentage).

b Kruskal–Wallis test or chi-squared test or Fisher’s exact test.

Statistically significant differences were observed between the phases for age and OT. Phase C patients were significantly older than those in Phase B (51.46 ± 9.28 years vs. 46.56 ± 7.29 years, *p* = 0.0016). Regarding OT, significant differences were noted between Phases A and B (66.17 ± 12.84 min vs. 54.32 ± 13.96 min, *p* = 0.0092) and between Phases A and D (66.17 ± 12.84 min vs. 54.5 ± 21.11 min, *p* = 0.0075). Additionally, OTs in Phase C were longer than in Phases B and D, although these differences did not reach statistical significance.

We further investigated potential differences in preoperative and perioperative characteristics between patients who experienced perioperative complications and those who did not (Table 3). In the “bladder injury” group, a significantly higher proportion of patients had no history of vaginal delivery (*p* = 0.022) and a history of at least one cesarean section (*p* = 0.045). However, no significant differences were observed in terms of BMI, age, specimen weight, OT, or surgical history.

**Table 3:** Comparison of clinical characteristics in patients with and without bladder injury^a^.

Variable	Bladder injury	No bladder injury	*p*^b^
*N*	5	134	
Age (years)	52.4 ± 11.52	48.9 ± 8.16	0.462
BMI (kg/m^2^)	25.32 ± 4.64	27.74 ± 5.21	0.246
History of vaginal delivery	1 (20)	102 (76.1)	0.022
History of cesarean section	3 (60)	23 (17.2)	0.045
History of laparoscopy	2 (40)	55 (41.0)	1.000
History of laparotomy	0	1 (0.7)	1.000
Operative time (minutes)	69.6 ± 24.48	58.94 ± 21.00	0.277
Weight of the pathology specimen after fixation (gram)	119.6 ± 95.38	263.80 ± 221.03	0.052

a Data are presented as mean ± standard deviation or as number (percentage).

b Mann–Whitney’s test or Fisher’s exact test.

## Discussion

Several publications describe the initial experiences of various centers with hysterectomies performed via V-NOTES (10–15). Our analysis of the learning curve using the CUSUM method identified four distinct phases. The first phase, the initiation phase, allows for the attainment of an initial proficiency level after approximately 12 hysterectomies. This aligns with the range reported in the literature, typically between 5 and 20 patients (10–15). The second phase, the consolidation phase, involves approximately 41 cases and consolidates mastery of the technique with a considerably reduced OT compared to the initiation phase. During this phase, the surgeon becomes more comfortable with the procedure, often leading to a gradual decrease in OTs. This phase demonstrates a steady improvement in performance as the surgeon’s skills are refined.

In contrast to other teams, our learning curve does not exhibit a plateau phase (10). Instead, we observed a transition directly into the third phase, the complexity phase. During this phase, OTs tend to increase again. Our analysis revealed that in this phase, the average age was significantly higher than in the previous phase, which may be attributed to a higher percentage of menopausal patients, resulting in more limited vaginal access. The remaining analyses did not show significant differences in other population characteristics. Although not statistically significant, it is noteworthy that in this phase, the average weight of the anatomic-pathological specimen was higher (304.89 ± 282.59 g), with uterine weights ranging from 30 to 1,271 g, compared to the initiation phase’s average weight of 206.08 ± 102.08 g. This may suggest an expansion of indications to include larger uteri than in the earlier phase. It is also noteworthy that this complexity phase marks the occurrence of the first perioperative complications (case 54), which required management and contributed to increased OT. Alternatively, it may be hypothesized that because our center is a training facility for assistants, the increase in OT during this phase could also be attributed to the surgeon’s involvement in teaching the technique and allowing less experienced surgeons to participate in the procedures. Our experience suggests that after performing approximately 53 cases, the surgeon attains sufficient proficiency to handle more complex cases and begin teaching and supervising the technique. The fourth phase, the mastery phase, is characterized by less variation in OTs, which stabilize around an average value.

Our rates of operative complications (3.6%) and postoperative complications (5%) are higher than those reported in the literature for V-NOTES hysterectomies (1.4% for operative complications and 3.8% for postoperative complications) (7), as well as for operative complications in laparoscopic hysterectomies (1.59%) (16). It is important to consider our complication rates in the context of our learning curve. Notably, the majority of complications occurred during phase C, the complexity phase. Compared to complication rates during the learning process of performing LH, studies report similar or even lower rates of perioperative complications (2.99%), postoperative complications (7.61%) (17), and overall complications (6.2%) (18). Notably, these studies describe the occurrence of ureteral and digestive injuries in laparoscopic hysterectomies, which were not observed in our series.

Analysis of our data identified two risk factors for bladder injury: a history of at least one cesarean section and the absence of a history of vaginal delivery. Other characteristics, such as age, BMI, surgical history, OT, or the weight of the surgical specimen, do not appear to influence the likelihood of bladder injury.

Our study has several limitations. First, it is a retrospective cohort study, which may introduce selection bias and affect the quality of the collected data. Additionally, although the results are based on a substantial number of cases, the study was performed in a single center with one experienced surgeon, potentially limiting the generalizability of the findings to other practitioners or clinical settings.

## Conclusion

This study allowed us to identify four distinct phases of skill development. The initiation phase encompasses the first 12 operations. After this phase, a consolidation phase of the technique is observed over the next 42 cases. During these two introductory phases, no perioperative complications were reported. We established that approximately 53 cases were necessary to achieve sufficient mastery of the technique, marking the transition into the third phase, known as the complexity phase. This phase did not definitively show differences in patient characteristics apart from age. However, we postulate that this phase entails instruction by the surgeon and observe a tendency towards heavier specimens. Ultimately, a fourth mastery phase is achieved after 107 cases, where OTs plateau around the mean OT.

Additionally, the analysis identified two risk factors for bladder damage: the absence of vaginal delivery and a history of cesarean section.

## Data Availability

The raw data supporting the conclusions of this article will be made available by the authors, without undue reservation.
